# The Accuracy and Reliability of Tooth Shade Selection Using Different Instrumental Techniques: An In Vitro Study

**DOI:** 10.3390/s21227490

**Published:** 2021-11-11

**Authors:** Nattapong Sirintawat, Tanyaporn Leelaratrungruang, Pongsakorn Poovarodom, Sirichai Kiattavorncharoen, Parinya Amornsettachai

**Affiliations:** 1 Department of Oral and Maxillofacial Surgery, Faculty of Dentistry, Mahidol University, Bangkok 10400, Thailand; nattapong.sir@mahidol.ac.th (N.S.); sirichai.kia@mahidol.ac.th (S.K.); 2 Education Program in Implant Dentistry, Faculty of Dentistry, Mahidol University, Bangkok 10400, Thailand; tanyaporn.lee@student.mahidol.edu; 3 Department of Prosthodontics, Faculty of Dentistry, Khon Kaen University, Khon Kaen 40000, Thailand; pongpoova@kkumail.com; 4 Department of Advanced General Dentistry, Faculty of Dentistry, Mahidol University, Bangkok 10400, Thailand

**Keywords:** shade selection, color determination, accuracy, photography, light, dental esthetics

## Abstract

This study aimed to investigate and compare the reliability and accuracy of tooth shade selection in the model using 30 milled crowns via five methods: (1) digital single-lens reflex (DSLR) camera with twin flash (TF) and polarized filter (DSLR + TF), (2) DSLR camera with a ring flash (RF) and polarized filter (DSLR + RF), (3) smartphone camera with light corrector and polarized filter (SMART), (4) intraoral scanner (IOS), and (5) spectrophotometer (SPEC). These methods were compared with the control group or manufacturer’s shade. The CIE Lab values (L, a, and b values) were obtained from five of the methods to indicate the color of the tooth. Adobe Photoshop was used to generate CIE Lab values from the digital photographs. The reliability was calculated from the intraclass correlation based on two repetitions. The accuracy was calculated from; (a) ΔE calculated by the formula comparing each method to the control group, (b) study and control groups were analyzed by using the Kruskal–Wallis test, and (c) the relationship between study and control groups were calculated using Spearman’s correlation. The reliability of the intraclass correlation of L, a, and b values obtained from the five methods showed satisfactory correlations ranging from 0.732–0.996, 0.887–0.994, and 0.884–0.999, respectively. The ΔE from all groups had statistically significant differences when compared to the border of clinical acceptance (ΔE = 6.8). The ΔE from DSLR + TF, DSLR + RF, SMART, and SPEC were higher than clinical acceptance (ΔE > 6.8), whereas the ΔE from IOS was 5.96 and all of the L, a, and b values were not statistically significantly different from the manufacturer’s shade (*p* < 0.01). The ΔE of the DSLR + RF group showed the least accuracy (ΔE = 19.98), whereas the ∆E of DSLR + TF, SMART, and SPEC showed similar accuracy ∆E (ΔE = 10.90, 10.57, and 11.57, respectively). The DSLR camera combined with a ring flash system and polarized filter provided the least accuracy. The intraoral scanner provided the highest accuracy. However, tooth shade selection deserves the combination of various techniques and a professional learning curve to establish the most accurate outcome.

## 1. Introduction

Aesthetics is one of the highest esthetic concerns and most challenging factors for dental practitioners [1,2,3]. To address the esthetic concern of the patient, a proper treatment plan, treatment technique, and communication is important. Tooth shade determination is the fundamental aspect of esthetic tooth restoration, especially in the anterior zone. Recently, technologies for color matching have been developed to increase the efficiency of esthetic restoration the accuracy, reproducibility, and communication. Current clinical techniques for tooth color determination in dentistry could be visual techniques and instrumental techniques.

Visual tooth color determination is the standard and the most generally used technique due to its simplicity and cost-effectiveness [4,5]. The major advantage of this technique is the ability to confirm the agreement of color between clinician and patient. However, the color determination accomplished by the visual method is usually applied by various standards, represents subjective outcomes, and mainly depends on the effectiveness of the human eyes. Despite the effectiveness of the human eye which can perceive a small color difference, the complexity of perception is not always infallible. Numerous factors need to be taken into account such as individual factors including observer’s age, sex, experience, color perception, eye fatigue, and environmental factors including the light source, location, and degree of light intensity [4,5,6,7].

The instrumental shade determination is developed to overcome the limitation of the visual method included individual and environmental factors. The literature reports the superiority of instrumental methods including intraoral scanners (IOS), spectrophotometers (SPEC), and digital cameras over visual methods [5,6,7,8,9,10]. Spectrophotometers are a high accuracy and reliable instrument for color matching in dentistry [4,8,11,12]. Frequently, they were chosen to be a referent device in many updated comparative studies [8,10,12]. However, because of its expensive cost, this device is not always available in routine dental practice.

Recently, digital technologies have been implemented in dentistry such as an intraoral scanner (IOS), 3D printing, etc. to enhance clinical success [13,14,15]. Some IOS such as 3Shape TRIOS (Copenhagen, Denmark) provide many new features to improve daily work basis. Systems are available with shade matching features which may lead to more convenience and lab communication support [8,9,14,16]. However, digital cameras and smartphone cameras have also been applied widely in the field of esthetic dentistry. A photograph taken from a digital single-lens reflex (DSLR) camera was claimed to provide accurate shade measurements with the conjunctive use of the software [17].

Munsell color space (L*C*h*: lightness, chroma, hue) is the color model widely used in dentistry and defines a color into hue, value, and chroma [8,17,18]. Hue is the color divided into 360 degrees [17]. The whites, blacks, and grays are indicated as achromatic or having no hue. The value shows the lightness of the color ranging from 0 for black to 100 for white [17,19]. Chroma can be described as the intensity or saturation of color represented in percentage where 0 represents no color and 100 is the full color [17,19]. Later, the Munsell color space system was upgraded into the Munsell Renovation System in which color can be expressed as a letter/number as evaluated using Munsell color charts [19,20]. Similarly, tooth shade can be revealed in hue, chroma, and value. In the dental field, hues are represented by the letters A, B, C, and D [21].

The L*a*b* color space or CIELAB is widely used for measuring object color in many industries [19,22]. Similar to L*c*h* space, L* refers to lightness ranging from 0 (black) to 100 (white). a* and b* refer to color directions: a* is the red–green color axis. (+a* is the red direction and −a* is the green direction) and b* is the yellow–blue color axis (+b* is the yellow direction and −b* is the blue direction) [19,22].

L*a*b* color space reveals a similar diagram as L*C*h color space; however, it uses rectangular coordinates instead of cylindrical coordinates [19]. It can be said that the L*a*b color space and the L*C*h color space are identical in the aspect of color distribution. Only the localization of the color points is calculated differently [22]. While the L* in both spaces remains the same, the conversion of L*a*b* color space into L*C*h color space can be done by the following formulas

(1) $$\mathrm{Chroma}\;\left(C\right)=\sqrt{a^{2}+b^{2}}$$

(2) $$\mathrm{hue}\;\left(h\right)=\;\sin\left(h\right)=\;b/\sqrt{a^{2}+b^{2}}$$

where, C = chroma, h = hue, and a and b = various color coordinates.

Color difference or delta E (ΔE) in the L*a*b* is used to quantify the color difference between two objects. Additionally, it is the widely used formula for color difference [23]. It represents in numerical value thus it can be objectively compared between specimens. The more of ∆E, the more color difference. ΔE is defined by the following formula:

(3) $$\Delta E\;=\sqrt{{\left(\Delta L\right)}^{2}+{\left(\Delta a\right)}^{2}+{\left(\Delta b\right)}^{2}}$$

In dentistry, ΔE can be expressed as acceptability threshold (AT) and perceptibility threshold (PT). The AT is the magnitude of color difference that is acceptable for esthetic outcomes [23] while the PT is the visually detectable color difference [23]. Thresholds for perceptible color mismatches have been reported to be significantly lower than thresholds for acceptability [11]. ΔE values of one can be detected by the human eye in the controlled environment with two objects placed next to each other [11,24]. Some studies reported, in the clinical situation, the human eye cannot detect the color difference unless the ΔE reaches 3.3 [11,25]. However, to Khashayar et al. [23], PT of ∆E = 1 and AT of ∆E = 3.7 is preferred.

There are various commercial shade guides widely used in dental practice. The VITA Classical shade guide (VITA Zahnfabrik, Bad Sackingen, Germany) is a common shade guide used in dentistry. The shade guide represents 16 natural shades based on the Vita chart and can be used in a single step of color matching. In addition, this shade guide was revealed to have consistent color amongst shade guides from the same manufacturer [4]. However, it is not able to present hue, chroma, and value individually [7]. The VITA 3D-Master shade guides (VITA Zahnfabrik, Bad Sackingen, Germany) are also reported to increase chances of success in tooth shade matching due to the resemblance of human tooth shade and allowing each dimension of color to be distinguished independently [7]. Moreover, it provides systems adapted to clinical application: Toothguide, Bleachedguide, and Linear guide.

The visual tooth color determination technique is based on the human eye which can discern very small color differences [4]. However, shade selection accomplished by the visual method is very subjective and mainly depends on the effectiveness of the human eyes. In addition, if an integrated shade feature in an intraoral digital scanner can substitute for other instrumental methods such as a spectrophotometer or digital photography is still unclear. Thus, this study aimed to investigate and compare the accuracy of shade selection via DSLR camera, smartphone camera, intraoral scanner, and spectrophotometer to the control group or manufacturer’s shade. Moreover, the reliability of shade selection among these various methods was also evaluated in this study.

## 2. Materials and Methods

### 2.1. Instrumentation

**Figure 1:**
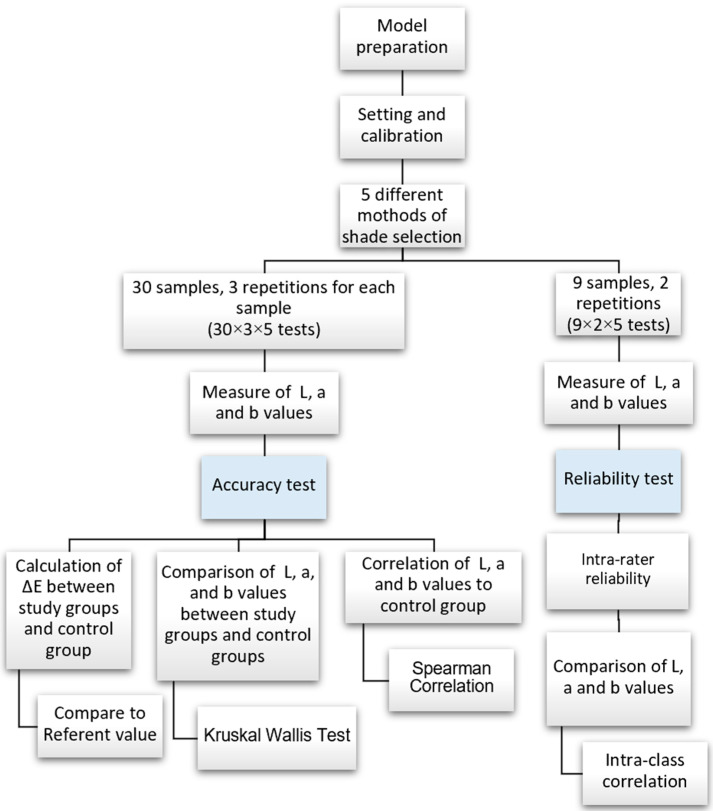
Overview of the study.

**Figure 2:**
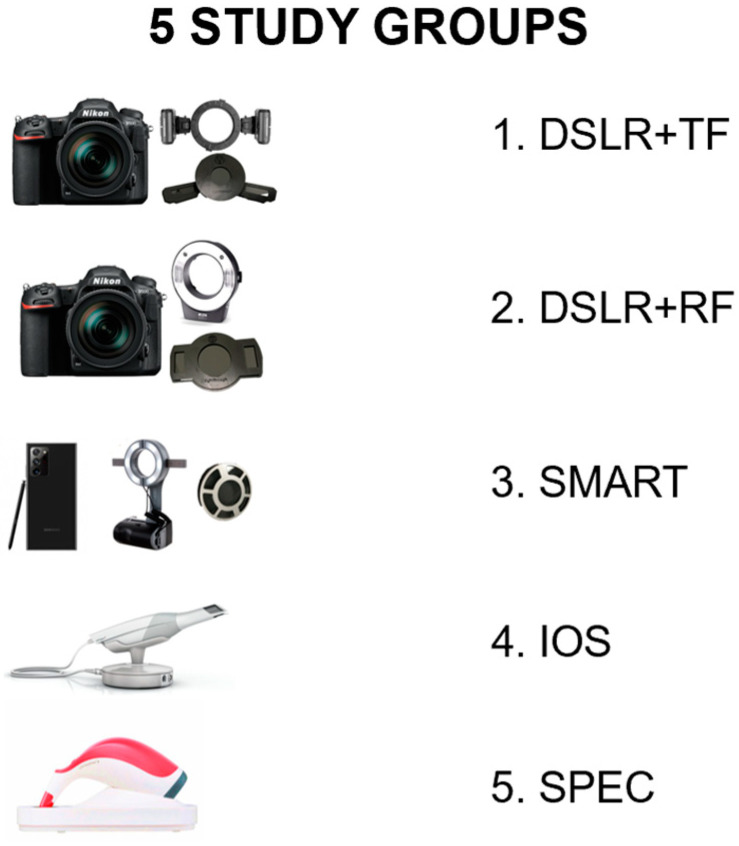
Study groups (shade selection methods) in this study.

This study was an in vitro experimental study. The overview of the study is shown in Figure 1 and the study groups of shade selection in this study are shown in Figure 2. Table 1 shows the various instruments and software used in this study. The sample size was calculated based on the standard deviation (SD) and mean from the previous study by Mehl and colleagues [16]. According to their study [16], the SD of ΔE values was reported as 2.58 and 5.09 in the study groups compared to 3Shape. Moreover, the mean was reported as 2.58 and 5.09 from the study groups. To estimate the sample size based on previous data by using the independent samples *t*-test under 80% power, 22 samples could be used for the accuracy testing. However, a sample size of 30 was taken in this present study for a more predictable outcome. Moreover, 2 repetitions were done on 9 different shades of crown for the reliability test. The reason for using 2 repetitions was based on the high reliability presented in the pilot study.

### 2.2. Model Preparation

**Figure 3:**
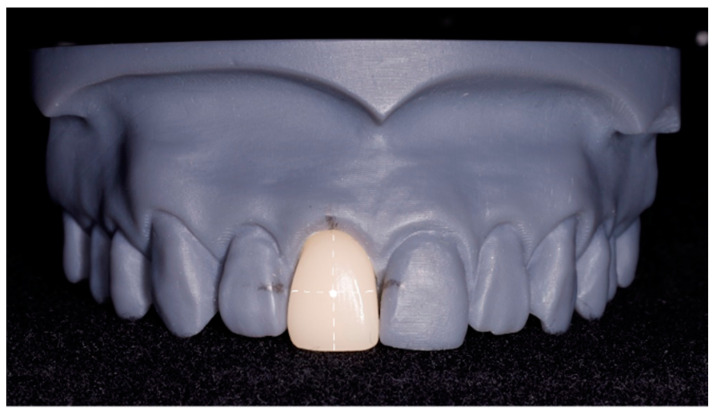
A gray resin model with milled right central incisor and the shade measuring point.

The model of the upper arch was made of resin extending from half of the first molar to half of the first molar. The 18% gray color of resin was selected to reduce the scattering light around the samples. The space of the right central incisor (tooth number 11) was prepared to replace by milled restorations fabricated by CAD/CAM. To fabricate the central incisor, first, the model was scanned by an intraoral scanner (Trios 3 Basic, 3Shape^®^, Copenhagen, Denmark). Second, the STL files were used to fabricate 30 milled restorations. Vitablocs^®^ mark II (VITA Zahnfabrik^®^, Bad Sackingen, Germany) which is a monochromic feldspar ceramic was utilized because of the various shade providence in the market. Nine possible VITA 3D-Master shades including 1M1, 1M2, 2M1, 2M2, 2M3, 3M1, 3M2, 3M3, and 4M2 were used to fabricate 30 teeth. The identical size and shape of those milled restorations were double-checked by the measurement from the vernier caliper. The tooth was retained in the model by friction to minimize the adjustment of the restoration and the referent points were marked. The cross-section of the imaginary lines, at the middle third of the tooth, was indicated as a shade measuring point (Figure 3).

### 2.3. Setting and Calibration

#### 2.3.1. Examiner

One examiner who was trained for shade matching by a specialist performed all the shade selection processes by various methods. Moreover, the examiner was blinded from manufacturer shade (control group) by letting an assistant prepare and set the order of the teeth separately.

#### 2.3.2. Digital Camera

Reproducibility of photography was mandatory. To conduct the least error, the environment, and the camera setting were well controlled. First, the environment; the experiment was in a closed room under a stable LED light. To have a more controlled light source, the mini-studio was set by using a foldable mini-studio. The main light source of the DSLR camera was from the flash system. For the smartphone camera, the main light source was from the light corrector with a 5500 K color temperature. Second, the camera setting; the camera setting was fixed for the repeatable process as shown in Table 2.

#### 2.3.3. Spectrophotometer

The spectrophotometer (VITA Easyshade^®^ Advance 5.0 (VITA Zahnfabrik, Bad Sackingen, Germany)) was calibrated each time before shade matching following the manufacturer’s recommendations. The device was set to the Vita 3D master mode of operation (Table 2).

#### 2.3.4. Intraoral Scanners

The software was updated to TRIOS 2014 software version 1.3.2.0 or later version. Before digital impression and tooth shade were determined, the calibration was done before scanning without the light source from the dental unit following the manufacturer’s suggestion (Table 2).

### 2.4. Shade Selection

**Figure 4:**
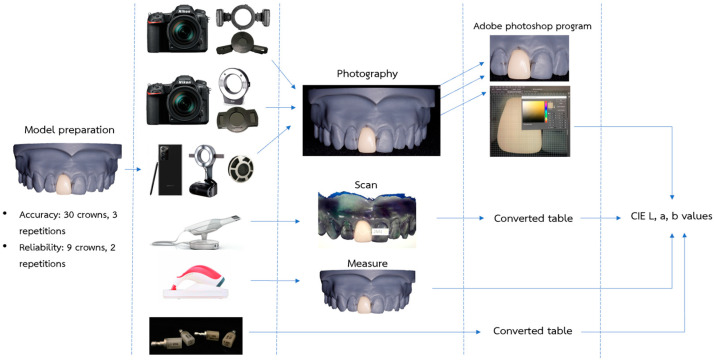
The details of the study. The CIE L, a, and b values were collected and used in data analysis.

All the instrumental shade matching methods were performed by an examiner using a DSLR camera, smartphone camera, intraoral scanner, and spectrophotometer, following Figure 4. All types of cameras were used with a polarized filter to decrease the reflection of the light.

#### 2.4.1. Digital Photography

Photographs were taken using the smartphone camera and DSLR camera under a foldable mini-studio (Foldio^®^, OrangeMonkie, Korea) with stable surrounding LED 5500 K light. A DSLR camera (Canon EOS RP, Canon^®^, Taichung Hsien, Taiwan) with a macro lens (Canon EF 100 mm F2.8L Macro IS USM) was used with two types of flash and their compatible polarized filters.

Firstly, the DSLR camera and twin flash (Macro twin lite flash MK-MT24, Meike global, Hongkong) were used with a polarized filter (LightThrough^®^, Bang Kapi, Thailand) for photography. The camera standardized parameters were adjusted before taking the photograph (Table 2). Cameras were stably placed 15 cm away from the sample. Photos were taken with a black background focusing on the referent point which is on the middle third of tooth 11. Moreover, a 5-s interval was applied while taking the photographs to allow stable flash intensity. Out of focus or blurred images were discarded and retaken to replace them. Thirty samples were taken with this method. Three repetitions for each sample were done. Therefore, a total of 90 photographs were selected for data collection in the accuracy test. For the reliability test, 9 samples representing 9 different shades were randomly selected for the analysis. Each sample needed 2 repetitions of the photograph. Thus, 18 photographs were taken and used for the reliability test. Therefore, a total of 108 photographs made via DSLR camera with twin flash were used for data collection.

Then the same methods were done using ring flash (Metz Mecabiltz ring flash MS 15, Metz, Germany) and polarized filter (LightThrough^®^, Bang Kapi, Thailand) for another set of photographs. (108 photographs for DSLR camera with ring flash group).

Finally, the smartphone camera was utilized with a light corrector (COCO Lux^®^, Hass, Korea) and its polarized filter. After that, the camera was set as in Table 2 and firmly placed before taking photographs. In this process, a total of 108 photographs were taken via the smartphone camera with a light corrector group.

**Figure 5:**
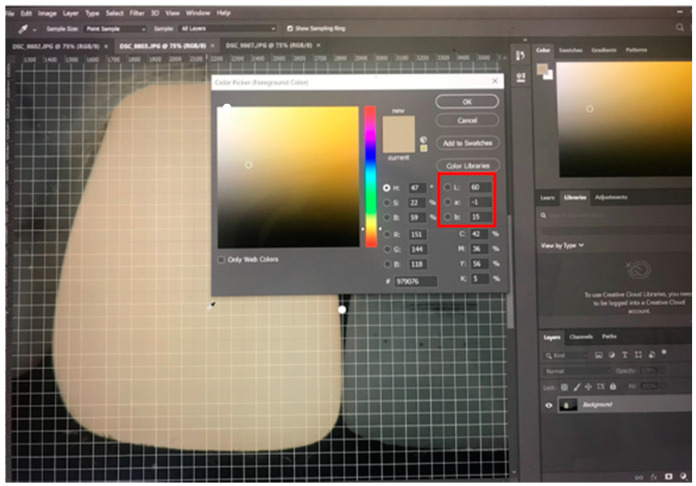
Data collection (L, a, and b value) on a cropped photograph in Adobe Photoshop 2020 for shade selection.

After photo data were collected, the total 324 RAW files were organized into 3 separate folders with a specific name for each method. The photographs were cropped, and Adobe Photoshop 2020 software was used to perform shade assessment (Figure 5). The point of measurement for each photograph was selected and the values of the CIE L*, a*, b* system, which is widely used in dentistry, were generated and compared between each method [8,17,18].

#### 2.4.2. Intraoral Scanner

**Figure 6:**
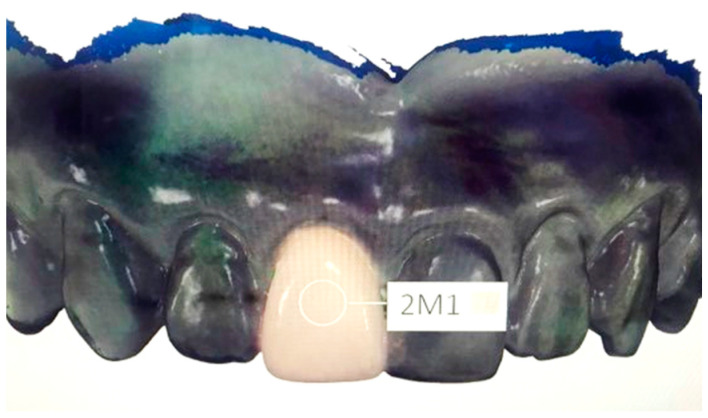
Shade selection with Trios 3 Basic. A shade measurement circle was placed on the buccal side at the middle third of the tooth.

The 3Shape TRIOS 3 basic intraoral scanner (3Shape^®^, Copenhagen, Denmark) was used in shade selection. After scanning the model and shade calibration following the manufacturer’s advice, the shade assessment was generated automatically when the examiner selected the shade selection feature and placed the shade measurement circle at the desired area. According to the manufacturer’s instruction, the circle was placed on the buccal side, and we avoided placing it at the interproximal or translucent area to generate the most accurate information. However, in this study, the circle was placed on the measuring point which was at the middle third of the tooth (Figure 6). The VITA 3D-Master shade value was obtained from the screen. Thirty samples were scanned and we assessed the shade value with this method. Three repetitions for each sample were made. Therefore, a total of 90 shade measurements were done for analysis in the accuracy test. Moreover, the 9 samples used for the reliability test were scanned and we measured each tooth shade 2 times. Therefore, the data from 108 shade measurements by intraoral scanner were obtained. Finally, the conversion table was used to convert the VITA 3D-Master shade value into three numerical data (L, a, and b values) [26].

#### 2.4.3. Spectrophotometer

The spectrophotometer used in this study was the VITA Easyshade Advance 5.0. (VITA Easyshade Advance 5.0, VITA Zahnfabrik, Bad Sackingen, Germany). After the instrument calibration following the manufacturer’s recommendations, the instrument was placed on the tooth with the same angle (90 degrees) at the middle of the tooth then shade assessment was automatically generated on the screen. The data were collected in L, a, and b values. A total of 90 assessments for the accuracy test on 30 teeth were made for calibration. Moreover, the 9 samples used for the reliability test were measured 2 times. Therefore, the data from 108 shade measurements were obtained. 

#### 2.4.4. Control Group

**Figure 7:**
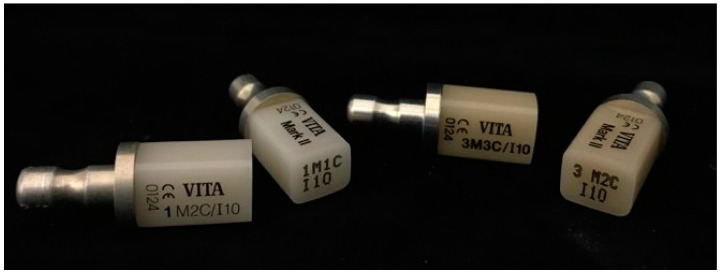
Vitablocs^®^ mark II 3D-master with the label of manufacturer’s shade on the block.

The manufacturer’s shade of Vitablocs^®^ mark II 3D-master was defined as the control group in this study. The shade values indicated under the standard of VITA Zahnfabrik were converted to the CIE Lab system using a converted table [26] as shown in Figure 7. Then, 3 numerical data were obtained from 30 crowns for data analysis.

The mean of L, a, and b values obtained by 5 methods including DSLR + TF, DSLR + RF, SMART, IOS, and SPEC were statistically compared to the control group for accuracy. Moreover, the mean L, a, and b values were also statistically analyzed for reliability. Finally, the ΔE or the color difference was calculated compared to the control group by using Equation (3).

### 2.5. Statistical Analysis 

The SPSS statistics program was utilized for statistical analysis. The numerical data of the CIE L, a, and b values were collected from 5 study groups and the control group.

The reliability was calculated from the intraclass correlation based on two repetitions. It was based on 18 tests per group (9 crowns × 2 repetitions).

The accuracy was calculated from; (a) ΔE calculated by the formula comparing each method to the control group, (b) study and control groups were analyzed by using the Kruskal–Wallis test, and (c) the relationship between study and control groups were calculated using Spearman’s correlation at a 95% significance level. One sample *t*-test was done to compare between the ∆E of study groups and the border of clinical acceptance (∆E = 6.8). The comparisons of CIE L, a, and b values between study groups and control groups were done using the Kruskal–Wallis test at a 95% significance level. The accuracy tests were based on 90 tests per group (30 crowns × 3 repetitions).

## 3. Results

### 3.1. Results of Reliability

The two repetitions of each method on nine different-shade crowns have been tested for reliability. According to intraclass correlation, the three numerical data of the CIE Lab system including L, a, and b values obtained from five methods ranged from 0.732–0.996, 0.887–0.994, and 0.884–0.999, respectively, at a 95% significance level. The further data of the intraclass correlation was also shown in Table 3.

### 3.2. Result of Accuracy

#### 3.2.1. Color Difference (ΔE)

The distribution of ∆E compared between study groups and control group is presented in Table 4. Only the IOS group was found to be in the range, while DSLR + RF represented the highest ΔE in this study.

The comparison of ∆E from study groups and the border of clinical acceptance (∆E = 6.8) by the one sample *t*-test is also presented in Table 5. All of the study groups had statistically significant differences to the border of clinical acceptance (∆E = 6.8). However, IOS presented the least mean difference (mean difference = 0.834).

#### 3.2.2. Kruskal–Wallis Test

For accuracy, 30 samples (*n* = 30) with 9 possible shades were accomplished. The distribution of CIE Lab values is presented in Table 6. Medians and *p*-values of CIE Lab values from 6 groups were presented by using the Kruskal–Wallis test at a 95% significance level.

It showed that L values obtained from all groups except IOS were statistically significantly different when compared to the control group at a 95% significance level (*p* < 0.01). Furthermore, statistically significant differences were found in a value for DSLR + RF and SMART when compared to the control group (*p* < 0.01). Finally, for the b value, only DSLR + RF had a statistically significant difference compared to the control group (*p* < 0.01) as shown in Table 7.

#### 3.2.3. Correlation of Various Methods with the Control Group

The descriptive data for Spearman’s correlation is shown in Table 6 and their comparison is shown in Table 8. Spearman’s correlation analysis showed that L values obtained from all groups were significantly correlated to the values of the control group, at a 95% significance level (*p*-value < 0.05). A stronger correlation was found for SMART (rho = 0.88) and SPEC (rho = 0.85) when compared to the other methods. Furthermore, significant correlations to the control group at a 95% significance level were found in a value for all groups except SPEC (rho = 0.11). Finally, the b value of all groups had a strong correlation (rho > 0.82) to the control group, at a 95% significance level as shown in Table 8.

## 4. Discussion

This study compared the tooth shade between various instrument techniques which were revealed the superior outcome in both accuracy and reproducibility compared to the visually conventional methods [5,7,11,27,28,29].

The natural tooth is not only polychromatic but also represents complex characteristics making shade selection more challenging. Dentin confers the based color of a dental element, or the hue, while the enamel modifies the chroma and the value of the hue according to its different thickness [21,30,31]. At the cervical third where the enamel is quite thin, the color tends to be darker than the middle and incisal thirds. It is suggested to use this area as a reference for hue selection [21]. The increasing of enamel thickness towards the middle third makes this area tend to represent less saturation of color [21]. Moreover, the difference in surface texture, the translucency of the tooth, and the color of the surrounding area influence tooth shade determination [7]. The surface texture of the tooth, both the macro-morphologic and micro-morphologic characteristics, affect the pattern of the light. Naturally, the light falling to the object could be reflected, absorbed, scattered, or transmitted [7]. It gives a diffuse reflection when the light contacts the gloss surface while giving a specular reflection when in contact with smooth surfaces [32]. It is important to understand that light scattering can affect color perception and shade determination [32]. In addition, the various degrees of enamel translucency and dentin opacity cause the layering effect on the tooth. This complexity of translucency makes color selection more challenging as it may fall outside of the color space in the dental shade guide [7].

In addition, an in vitro study was designed due to the aim of using true shade verification. The advantage of using the manufacturer’s shade as a control group is to refer to “accuracy” as the color’s trueness. Precisely, Mehl and colleagues were concerned about the use of “accuracy” in various in vivo studies because they believed that the true shades of natural teeth were not exactly known [10,16]. Therefore, they preferred to use “relative accuracy” in their study instead. 

This present study was performed in five study groups, including (1) DSLR + TF, (2) DSLR + RF, (3) SMART, (4) IOS, and (5) SPEC. The CIE Lab values (L, a, and b values) of all groups showed satisfying results for intraclass correlation (r > 0.7, r > 0.8, and r > 0.8, respectively). Although two repetitions were performed, the reliability test showed an optimal outcome. More precisely, all those instruments used in this study provided high reliability of tooth shade selection.

From the accuracy point of view, the DSLR camera with ring flash and polarized filter (DSLR + RF) had the least accuracy as the null hypothesis was rejected for all CIE Lab values (L, a, and b values) when compared to the manufacturer’s shade. (*p* < 0.01, *p* < 0.01, and *p* < 0.01, respectively). Moreover, the highest ΔE (∆E = 19.98) was shown in this group when compared to all methods. Delta E (∆E) or color difference is used to quantify the color difference between two objects. The more ∆E, the more color unacceptability. Sampio and colleagues reported the DSLR camera with ring flash group and the iPhone 7 represented the highest ΔE when compared to a DSLR camera with various types of twin flashes [33]. This study represents beneficial data on the accuracy of tooth shade selection by photography using digital cameras with different kinds of techniques and equipment. However, there was no comparison of using a DSLR camera with ring flash and polarized filter or an adjustable smartphone camera with the polarized filter.

Based on the result from this present study, using an IOS showed the highest accuracy due to all of CIE Lab values (L, a, and b values) not having statistically significant differences when compared to the manufacturer’s shade (*p*-value = 1, *p*-value = 0.5, and *p*-value = 1, respectively). Moreover, this group presents the lowest ΔE when compared to all groups (∆E = 5.96) and it was in the range of estimated clinical acceptance (ΔE < 6.8) [34], while three groups including SMART, DSLR + TF, and SPEC showed similar ΔE (10.57, 10.90, and 11.57 respectively), and all of them were higher than the estimated clinical acceptance. This means, from this study, only shade determination from the IOS group represented an acceptable esthetic outcome.

The reasons that support the intraoral scanner having the highest accuracy can be discussed in three topics. First, among the three color dimensions, the L value showed the most variability in this study. L values obtained from all groups except IOS were statistically significantly different when compared to the control group at a 95% confidence level (*p*-value < 0.01). The L value in CIE Lab color space is referred to as lightness. It was shown to be the easiest detectable color dimension if the mismatch had occurred. The literature supported that value dimension or lightness showed to be the most important dimension as it allows the achievement of esthetic outcome [7]. According to the result, this might be the reason why the IOS group represented the best accuracy when compared to other groups.

Second, with the process of scanning and the basic system of intraoral scanners supported by a high-definition camera with LED, the outer light source may have less effect on them, especially when compared to digital camera groups. 

Lastly, due to the various instrumental techniques used in this study, the converted table was applied to be a part of the method when using the intraoral scanner. Therefore, the data gained from this group was revealed to be less varied compared to other groups.

From the digital photography point of view, over the years, the digital camera has been beneficially promoted for tooth shade selection in esthetic dentistry. The evidence has outlined that digital photography provided easy accessibility, better laboratory communication, and great intraoral information for tooth color and characteristics [5,17,33,35]. Moreover, using the cross-polarized filter provides a beneficial outcome by eliminating or reducing the effect of scattering light from the environment, which interferes with the color measurements [33,36]. However, care must be taken on the professional learning curve of using a digital camera, techniques, associated equipment, and software-generated color to ensure the best accuracy and reliability of tooth shade selection [5].

According to digital photography, the Kruskal–Wallis test showed the best accuracy on DSLR + TF then followed by SMART and DSLR + RF, respectively. Precisely, DSLR + TF showed the best accuracy because only the L value had the statistically significant difference when compared to the control group. On the other hand, SMART and DSLR + RF represented statistically significant differences to two and three values, respectively. However, the ΔE of SMART and DSLR + TF were similar (ΔE = 10.57 and 10.90 respectively). Sampio and colleagues in 2019 mentioned the use of a DSLR camera with closed-up twin flash and the polarized filter was the most accurate due to the lowest ΔE (3.4 ± 1.0) [33]. While using a DSLR camera with ring flash and iPhone 7 camera without polarized filter had similar ΔE and showed the highest ΔE (7.5 ± 5.7 and 7.5 ± 3.9 respectively) when compared to different types of twin flash [33]. It is confirmed that a different pattern of light from the various flash systems affects color perception. However, the evidence of tooth shade selection assisted by digital photography with a variety of flash systems is still scarce. According to the smartphone camera, even if it is one of the top models in the market that provides high image resolution, it appears to be inferior to a DSLR camera. For more flexible parameter adjustment, Tam and lee suggested using a customized mode of smartphone camera instead of automatic white balance in an unstable light [37]. Additionally, the clinical study confirmed avoiding automatic white balance mode from a smartphone camera for tooth shade selection because of the color compensation. Not only were the yellow shades from the tooth compensated by the blue color but also the red shades from the gingiva were compensated by the blue-green color [38]. However, using the customized adjustable smartphone camera with constant illumination and the polarized filter is a further step in future dental photography and shade selection. The smartphone cameras provide various superior characteristics such as small size, lightweight, easy accessibility, and user-friendly [33,37]. Therefore, it could be a potential alternative to other tooth shade matching procedures.

Reyes and colleagues confirmed in vivo study that the repeatability of 3Shape TRIOS intraoral scanner (86.66%) is superior to visual matching (75.22%) [7]. This result is supported by previous studies [7,8,9,16]. In addition, it was reported that chroma was the color dimension that demonstrated the lowest repeatability in both methods; the visual method and TRIOS intraoral scanner [7].

The spectrophotometers have high accuracy and reliable instrument for color matching in dentistry [4,8,11,12]. Some in earlier studies were designed to use them as a referent device for shade matching [8,10,12]. Rutkunas and colleagues in 2019 had chosen Spectroshade which is a complete tooth surface measurement spectrophotometer as a referent device in their in vivo study. Spectroshade was claimed to be a reliable and precise device [12]. However, this study was designed for in vitro study and was able to compare SPEC (VITA Easyshade^®^ Advance 5.0) with the manufacturer’s shade. According to the result, SPEC revealed a statistically significant difference only in L value (*p* < 0.01). However, the ΔE was over than the estimated clinical acceptance (ΔE = 11.57). This could be explained by the edge loss effect that could happen to the point measurement spectrophotometer. This type of device cloud limits the area of color analysis only by 3–5 mm around the tip due to its small aperture size [4]. Additionally, a study found a negative influence on accuracy and reliability when using Easyshade with freehand approach while it did not reveal in Spectroshade [39]. Moreover, Spectroshade was reported to be more reliable than Easayshade in a clinical situation [40]. In contrast, the literature revealed VITA Easyshade was more accurate (92.6%) in tooth color matching compared to Spectroshade (80.2%) [11].

It has been seen that the referent shade guide system is related to the accuracy and reliability of shade determination. In this study, the VITA 3D-Master shade scale was used not only because of the wider range and distribution of color but also the superior result according to accuracy and reliability when compared to the VITA Classic shade guide. Liberato and colleagues concluded that the VITA 3D-Master shade scale showed superior agreement than the VITA Classic shade guide [6]. They also showed in the result that the VITA Classic shade guide gave less reliability than the VITA 3D-Master shade guide [6]. In the in vivo study, likewise, the accuracy of color matching in the intraoral scanner was better with the VITA 3D-Master shade guide (53.3%) than the VITA Classic shade guide (27.5%) [12].

With this limitation of in vitro studies, some related factors must be taken into account when applied intraorally, for instance, saliva, light, the polychromatic shade of the natural tooth, the camera fixation and distance, etc. Moreover, the converted table was applied to be a part of the method when using the intraoral scanner. Therefore, the variety of data from this group could be limited. Further studies should be concerned for better data conversion when using different instrumental techniques. Moreover, the attempt related to dental photography should add a gray card to neutralize image colors with more accurate software for better shade matching. For the most accurate outcome, the combination of many techniques and a learning curve for dental practitioners are required.

## 5. Conclusions

The reliability of five different methods; (1) digital single-lens reflex (DSLR) camera with twin flash (TF) and polarized filter (DSLR + TF), (2) DSLR camera with a ring flash (RF) and polarized filter (DSLR + RF), (3) smartphone camera with light corrector and polarized filter (SMART), (4) intraoral scanner (IOS), and (5) spectrophotometer (SPEC) was compared with the control group or manufacturer’s shade. The DSLR camera combined with a ring flash system and polarized filter provided the least accurate and significant differences of all CIE Lab values when compared to manufacturer shades. The intraoral scanner provided the highest accuracy and all of the CIE Lab values did not have significant differences when compared to the manufacturer’s shade. Hence, the tooth shade selection deserves the combination of various techniques and a professional learning curve to establish the most accurate outcome.

## Figures and Tables

**Table 1:** Various instruments and software used in this study.

Equipment	Description	Manufacturer
30 blocks of Vitablocs^®^ mark II 3D-master	Monochromatic, tooth-colored feldspar ceramic. Size I10 for central incisor and 9 different shades including 1M1, 1M2, 2M1, 2M2, 2M3, 3M1, 3M2, 3M3, and 4M2	VITA Zahnfabrik^®^, Bad Sackingen, Germany
VITA Easyshade^®^ Advance 5.0	Spectrophotometer	VITA Zahnfabrik^®^, Bad Sackingen, Germany
Nikon D500	DSLR camera	Nikon^®^, Tokyo, Japan
Macro twin lite flash MK-MT24	Twin flash	Meike global^®^, Hongkong
Metz Mecabiltz ring flash 15MS-1	Ring flash	Metz^®^, Zendorf, Germany
AF-S Micro Nikkor 105 mm f/2.8G ED	Macro lens	Nikon^®^, Tokyo, Japan
LightThrough for Metz^®^ ring flash	Polarized filter for ring flash	LightThrough^®^, Bang Kapi, Thailand
LightThrough for Meike twin flash	Polarized filter for twin flash	LightThrough^®^, Thailand
Galaxy Note 20 Ultra 5G camera	Smartphone camera	Samsung C&T^®^, Seoul, Korea
Adobe^®^ Photoshop^®^ 2020 program	Image editing software generated the CIE L*a*b* value for photographs	Adobe^®^, San Jose, CA, USA
Trios 3 Basic	Intraoral scanner	3Shape^®^, Copenhagen, Denmark
Smartphone light corrector	External light source for smartphone	COCO Lux^®^, Hass, Gyeonggi-do, Korea
Foldable mini-studio	Mini-studio for photography	Foldio^®^, OrangeMonkie, San Diego, CA, USA

**Table 2:** Calibration of each method before the experiment.

Group	Photo	Equipment	Parameter Calibration
1	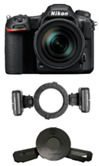	DSLR camera with twin flash and polarized filter: DSLR + TF	Exposure (1/250 s, f32), ISO (100), flash (ETTL), magnification ratio (1:1), white balance (Auto) distance (30 cm)
2	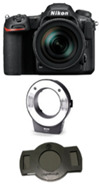	DSLR camera with ring flash and polarized filter: DSLR + RF	Exposure (1/250 s, f32), ISO (100), flash (ETTL), focusing ratio (1:1), white balance (Auto), distance (30 cm)
3	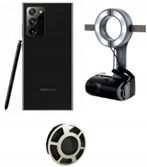	Smartphone camera with light corrector and polarized filter: SMART	Exposure (f50), ISO (200), flash (Auto), focusing ratio (1:1), white balance (5000 K), distance (15 cm), light corrector’s mode (ring mode)
4	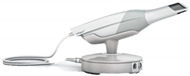	Intraoral scanner: IOS (Trios 3)	Calibrate following the manufacturer’s recommendations before used, Software: TRIOS 2014 software–1.3.2.0 or later
5	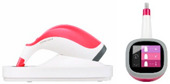	Spectrophotometer: SPEC	Calibrated following the manufacturer’s recommendations each time, Mode: Vita 3D master

**Table 3:** Reliability: the intraclass correlation of CIE Lab values from study groups.

Measurements	Group	95% Confidence Interval		Mean	Intraclass Correlation
		Lower Bound	Upper Bound		
L value	DSLR + TF	0.482	0.963	0.917	0.847
	DSLR + RF	0.341	0.976	0.939	0.886
	SMART	0.225	0.931	0.845	0.732
	IOS	0.986	0.999	0.998	0.996
	SPEC	0.955	0.997	0.994	0.989
a value	DSLR + TF	0.670	0.983	0.960	0.923
	DSLR + RF	0.573	0.974	0.940	0.887
	SMART	0.766	0.987	0.970	0.943
	IOS	0.977	0.999	0.997	0.994
	SPEC	0.789	0.998	0.994	0.987
b value	DSLR + TF	0.892	0.994	0.986	0.973
	DSLR + RF	0.935	0.996	0.992	0.985
	SMART	0.991	1.000	0.999	0.998
	IOS	0.994	1.000	0.939	0.884
	SPEC	0.597	0.972	0.999	0.999

DSLR + TF: digital single-lens reflex camera with twin flash and polarized filter, DSLR + RF: DSLR camera with a ring flash and polarized filter, SMART: smartphone camera with light corrector and polarized filter, IOS: intraoral scanner, SPEC: spectrophotometer.

**Table 4:** The distribution of the color difference (ΔE).

Group	Minimum	Maximum	Mean	SD
DSLR + TF	5.78	14.82	10.90	2.29
DSLR + RF	15.65	25.45	19.98	2.66
SMART	6.74	16.80	10.57	2.36
IOS	3.40	9.35	5.96	1.83
SPEC	7.93	14.54	11.57	1.53

**Table 5:** Accuracy: one sample *t*-test used to compare between ∆E from study groups and the border of clinical acceptance (∆E = 6.8).

Measurements	Comparison Groups		t	Mean Difference	*p* Value
∆E	∆E = 6.8	DSLR + TF	9.814	4.106	<0.01
		DSLR + RF	27.106	13.181	<0.01
		SMART	8.755	3.775	<0.01
		IOS	−2.496	0.834	0.018
		SPEC	17.098	4.776	<0.01

DSLR + TF: digital single-lens reflex camera with twin flash and polarized filter, DSLR + RF: DSLR camera with a ring flash and polarized filter, SMART: smartphone camera with light corrector and polarized filter, IOS: intraoral scanner, SPEC: spectrophotometer. Comparison was done using the one sample *t*-test. Significant difference at *p*-value < 0.05.

**Table 6:** The distribution of CIE Lab values from all groups.

Measurements	Groups	Mean	SD	Min	Max
L value	DSLR + TF	58.30	2.18	53.00	61.00
	DSLR + RF	53.33	3.05	48.00	61.00
	SMART	60.66	2.52	56.00	65.00
	IOS	72.99	4.48	64.40	83.10
	SPEC	79.53	2.69	74.90	84.50
	Control	68.73	3.69	62.60	75.20
a value	DSLR + TF	2.00	2.55	−1.00	7.00
	DSLR + RF	−0.900	1.24	−5.00	2.00
	SMART	4.57	1.98	2.00	9.00
	IOS	1.89	0.99	−1.00	4.20
	SPEC	1.16	0.60	0.10	2.20
	Control	0.96	1.13	−0.50	3.40
b value	DSLR + TF	18.83	4.73	12.00	25.00
	DSLR + RF	5.60	4.03	0.00	12.00
	SMART	13.43	6.95	4.00	22.00
	IOS	16.10	2.33	12.50	21.10
	SPEC	18.19	7.18	9.20	26.90
	Control	18.00	4.10	10.80	24.70

SD: standard deviation, Min: minimum, Max: maximum. DSLR + TF: digital single-lens reflex camera with twin flash and polarized filter, DSLR + RF: DSLR camera with a ring flash and polarized filter, SMART: smartphone camera with light corrector and polarized filter, IOS: intraoral scanner, SPEC: spectrophotometer.

**Table 7:** The accuracy: Kruskal–Wallis test of CIE Lab values between 5 test groups and control groups.

Measurements	Comparison Groups		Mean Difference	*p*-Value
L value	Control	DSLR + TF	10.43	<0.01
		DSLR + RF	15.4	<0.01
		SMART	8.07	<0.01
		IOS	4.26	1.00
		SPEC	10.8	<0.01
a value	Control	DSLR + TF	1.04	1.00
		DSLR + RF	1.86	<0.01
		SMART	3.54	<0.01
		IOS	0.93	0.50
		SPEC	0.2	1.00
b value	Control	DSLR + TF	0.83	1.00
		DSLR + RF	12.4	<0.01
		SMART	4.57	0.23
		IOS	1.9	1.00
		SPEC	0.19	1.00

DSLR + TF: digital single-lens reflex camera with twin flash and polarized filter, DSLR + RF: DSLR camera with a ring flash and polarized filter, SMART: smartphone camera with light corrector and polarized filter, IOS: intraoral scanner, SPEC: spectrophotometer. Comparison was done using the Kruskal–Wallis Test. Significant difference at *p*-value < 0.05.

**Table 8:** Correlation of various methods with the control group.

Groups	L Value	a Value	b Value
DSLR + TF			
rho	0.64	0.66	0.89
*p* value	<0.01	<0.01	<0.01
DSLR + RF			
rho	0.71	0.57	0.91
*p* value	<0.01	<0.01	<0.01
SMART			
rho	0.88	0.74	0.93
*p* value	<0.01	<0.01	<0.01
IOS			
rho	0.38	0.63	0.82
*p* value	0.038	<0.01	<0.01
SPEC			
rho	0.85	0.11	0.92
*p* value	<0.01	0.56	<0.01

DSLR + TF: digital single-lens reflex camera with twin flash and polarized filter, DSLR + RF: DSLR camera with a ring flash and polarized filter, SMART: smartphone camera with light corrector and polarized filter, IOS: intraoral scanner, SPEC: spectrophotometer. Comparison was done using Spearman’s correlation. Significant difference at *p*-value < 0.05.

## Data Availability

The data presented in this study are available on request from the corresponding authors.
